# Chronology of reproductive investment determines predation risk aversion in a felid‐ungulate system

**DOI:** 10.1002/ece3.4947

**Published:** 2019-02-26

**Authors:** Daniel A. Crawford, Michael J. Cherry, Brian D. Kelly, Elina P. Garrison, David B. Shindle, L. Mike Conner, Richard B. Chandler, Karl V. Miller

**Affiliations:** ^1^ Warnell School of Forestry and Natural Resources University of Georgia Athens Georgia; ^2^ College of Natural Resources and Environment Virginia Polytechnic Institute and State University Blacksburg Virginia; ^3^ Fish and Wildlife Research Institute Florida Fish and Wildlife Conservation Commission Gainesville Florida; ^4^ U.S. Fish and Wildlife Service Immokalee Florida; ^5^ Joseph W. Jones Ecological Research Center Newton Georgia

**Keywords:** behavioral risk effect, predation risk, reproductive chronology, risk aversion, sexual segregation

## Abstract

1. Fear of predators can behaviorally mediate prey population dynamics, particularly when predation risk influences reproductive investment. However, the costs of reproductive investment may mitigate predation risk aversion relative to periods when the link between reproductive output and prey behavior is weaker.
2. We posit that intensity of reproductive investment in ungulates may predict their response to predation risk such that the sexes increase risk exposure during biological seasons that are pivotal to reproductive success, such as the fawn‐rearing and breeding seasons for females and males, respectively.
3. We examined the activity patterns of sympatric white‐tailed deer (*Odocoileus virginianus*), a sexually segregated polygynous ungulate, and Florida panthers (*Puma concolor coryi*) in the context of the “risky times – risky places hypothesis” and the reproductive strategy hypothesis. We compared detection rates and diel activity overlap of both species using motion‐triggered camera traps positioned on (*n* = 120) and off (*n* = 60) anthropogenic trails across five reproductive seasons.
4. Florida panthers were nocturnal and primarily observed on‐trail providing an experimental framework with risky times and risky places. Contrary to studies in other taxa inversely correlating prey reproductive investment to predation risk, the sexes of deer were more risk prone during sex‐specific seasons associated with intense reproductive investment.
5. Our results suggest spatiotemporally variable predation risk influences sex‐specific behavioral decision‐making in deer such that reproductive success is maximized.

## 1 INTRODUCTION

Predators impact prey populations directly through mortality or indirectly through nonconsumptive predation risk effects. Predators may induce morphological shifts in prey. Physiological responses of prey to risk of predation also affect prey demography as elevated risk of predation can suppress reproductive rates and decrease survival by altering metabolic processes (Clinchy, Zanette, Boonstra, Wingfield, & Smith, 2004; Sheriff, Krebs, & Boonstra, 2009; Travers, Clinchy, Zanette, Boonstra, & Williams, 2010; Zanette, White, Allen, & Clinchy, 2011). Furthermore, prey populations may experience residual physiological effects in the absence of predators as a result of maternal programming (Sheriff, Krebs, & Boonstra, 2010; Storm & Lima, 2010). In addition to affecting morphology and physiological processes, predators also influence prey behavior as individuals attempt to optimize foraging such that energetic intake is maximized and risk is minimized. Brown, Laundré, and Gurung (1999) reviewed behavioral effects of predation risk such as shifts in space use, temporal activity patterns, and rates of vigilance, and suggested that such effects pervade behaviorally responsive predator–prey systems. To fully understand the interactions between predators and their prey, it is important to consider the cumulative impacts of both consumptive and nonconsumptive interactions.

Animals must balance energy and activity budgets (Lima, 1998), particularly when energetically profitable forage patches also impose the greatest risk of predation (Werner & Anholt, 1993). In these situations, animals must decide when, where, how, and how long to forage based on their assessment of the risk of predation (Lima & Dill, 1990). Brown et al. (1999) describe the “ecology of fear” as a framework for understanding the trade‐offs associated with behavioral decision‐making in prey species. By expanding optimal foraging theory to include predation risk, they propose a classification scheme that delineates N‐driven (mortality driven) from µ‐driven (behaviorally driven) predator–prey systems (Lima & Dill, 1990). Laundré, Hernandez, and Altendorf (2001) expand upon this notion by conceptualizing the “landscape of fear” and explaining how predation risk can spatially structure communities. However, perception of risk is context‐dependent such that stage‐ or sex‐specific requirements of the prey species may lend to demographic variation in behavioral decision‐making.

Sexually dimorphic polygynous ungulates often exhibit sex‐specific variation in behavioral decision‐making (Barboza & Bowyer, 2000). Many hypotheses attempt to explain the behavioral differences between the sexes, but a generalizable consensus regarding drivers of demographically variable behavior is lacking as a result of the context‐specific factors (i.e., predator community, habitat composition) affecting ungulate species (Bleich, Bowyer, & Wehausen, 1997; Festa‐Bianchet, 2012; Pérez‐Barbería, Robertson, & Gordon, 2005). Despite variability in behavior associated with species‐ and site‐specific scenarios, male body size is ubiquitously correlated with breeding success and female nutritional status with maternal investment in offspring (Hamel, Côté, Gaillard, & Festa‐Bianchet, 2009). Male breeding success and maternal investment are cornerstones of fitness; however, predation risk must be considered to understand ungulate fitness as both sexes attempt to optimize energetic intake under risk of predation according to their respective reproductive physiologies and energetic demands. Thus, risk of predation may result in sex‐specific behavioral decisions that differentially impact the relative fitness of males and females. Further, relative paternal and maternal investment in offspring should contribute to sexual divergence in behavioral decision‐making because females bear sole responsibility for rearing offspring.

White‐tailed deer (*Odocoileus virginianus*; hereafter deer) are a model species for behavioral investigations of sexually dimorphic polygynous ungulates. The species’ expansive range and high abundance have afforded investigators the opportunity to study antipredator behaviors in the context of region‐specific factors such as climate, habitat, and predator community. From the boreal forests of Canada populated by wolves (*Canis lupus*) to the tropical rainforests of South America inhabited by puma (*Puma concolor*) and jaguar (*Panthera onca*), a robust literature describes antipredator responses including grouping behavior, flight distance, alarm signaling, vigilance, giving‐up densities, and shifts in space use (Brown, 1999; Cherry, Conner, & Warren, 2015; Hirth & McCullough, 1977; LaGory, 1987; Lashley et al., 2014; Lingle, 2001; Messier & Barrette, 1985; Rieucau, Vickery, & Doucet, 2009). However, little is known about how deer behaviorally negotiate variability of predation risk across the landscape at both diel and seasonal time scales. Understanding spatiotemporal behavioral responses to predation risk is further complicated by predator‐specific traits that affect the magnitude of response in prey. For example, ambush predators are predicted to induce risk effects of greater magnitude than active, cursorial predators due to the association of the predator with habitat cues (Preisser, Orrock, & Oswald, 2007).

The restoration of the endangered Florida panther (*P. c. coryi*; hereafter panther), an efficient ambush predator of adult deer, in southwestern Florida provides an opportunity to investigate behavioral responses of deer to predation risk that varies in space and time. Since 1995, the panther population increased 14% annually from an estimated 20–25 to an estimated 100–180 independent individuals by 2016 (Johnson et al., 2010; Florida Fish & Wildlife Conservation Commission, 2016). The recovery of large carnivores has been shown to induce shifts in ungulate behavior (Berger, Swenson, & Presson, 2001; Creel, Winnie, Maxwell, Hamlin, & Creel, 2005; Kauffman et al., 2007; Laundré et al., 2001). Middleton et al. (2013) documented relatively weak risk effects of wolves on elk (*Cervus elaphus canadensis*) in the Greater Yellowstone Ecosystem following restoration of the cursorial predator. However, no attention has been given to risk effects associated with the restoration of an ambush predator. Furthermore, few studies have employed the use of remote‐sensing cameras distributed at high densities and broad distribution to concurrently monitor predator and prey.

We examined the effects of variation in panther predation risk across space and at multiple temporal scales on activity patterns of male and female deer. We tested the hypothesis that predation risk would induce sex‐specific differences in spatiotemporal activity patterns as determined by spatial (“risky places hypothesis”) and temporal (“risky times hypothesis”) variation in panther activity. High‐risk scenarios were characterized by relatively high panther activity. We hypothesized that risk proneness would increase with the relative reproductive importance of each biological season to each sex. Under this hypothesis, females investing in lactation during the fawn‐rearing season should increase their predisposition to risk relative to other, less demanding seasons (Oftedal, 1985). Because male reproductive success is positively correlated with body mass (DeYoung, Demarais, Honeycutt, Gee, & Gonzales, 2006; Townsend & Bailey, 1981), they should be more risk prone than females across all biological seasons. Specifically, we predicted that males would be most risk prone during the breeding season.

## 2 METHODS

### 2.1 Study area

The Big Cypress Basin (BCB) of southwestern Florida is characterized by a seasonal tropical climate with hot summers accounting for more than 60% of annual rainfall and relatively dry, mild winters creating distinct wet and dry seasons (Duever, 1986; Harlow, 1959; Loveless, 1959; McPherson, 1974). Mean daily temperatures ranged from 14 to 28°C with an annual mean temperature of 23°C (Duever, 1986). Minimal relief characterized regional topography with slight ridges delineating relatively flat basins interspersed with depressions that retain standing water throughout the dry season (Duever, 1986; McPherson, 1974). A 9 cm/km slope to the southwest induced a southwestern sheet flow of water across the landscape. Low relief along with warm season precipitation characteristic of the regional climate contributed to seasonal inundation of much of the landscape with mean water depths ranging from 0.3 to 0.73 m.

Five vegetation communities dominated the BCB including pine flatwoods, hardwood hammocks, cypress swamps, prairies, and marshes. Pine flatwoods forests are dominated by slash pine (*Pinus elliottii*) with an understory of cabbage palm (*Sabal palmetto*), saw palmetto (*Serenoa repens*), and hardwood shrubs. Ground cover in pine flatwoods consisted primarily of grasses. Hammock forests existed on areas of higher elevation and consisted of hardwoods, palms, ferns, and shrubs (McPherson, 1974). Cypress communities existed at lower elevations and varied in composition from open stands of cypress (*Taxodium distichum*) varying in size with minimal herbaceous growth interspersed to mixed swamps with dense tangles of trees, vines, shrubs, and epiphytes and are indicative of drainage areas (Harlow, 1959; Harlow & Hooper 1971; Duever, 1986). Both types of prairie, wet and dry, consisted of grasses with few trees; however, wet prairies included a mixture of prairie and marsh communities. Lastly, emergent wetland vegetation such as sawgrass (*Cladium mariscus*) and rushes (*Juncus *spp.) dominated marshes with alligator flag (*Thalia geniculata*) dominating deeper depressions. Typical water depths in marshes exceeded that of surrounding wet prairies and cypress communities by several centimeters (Harlow & Hooper, 1971).

**Figure 1:**
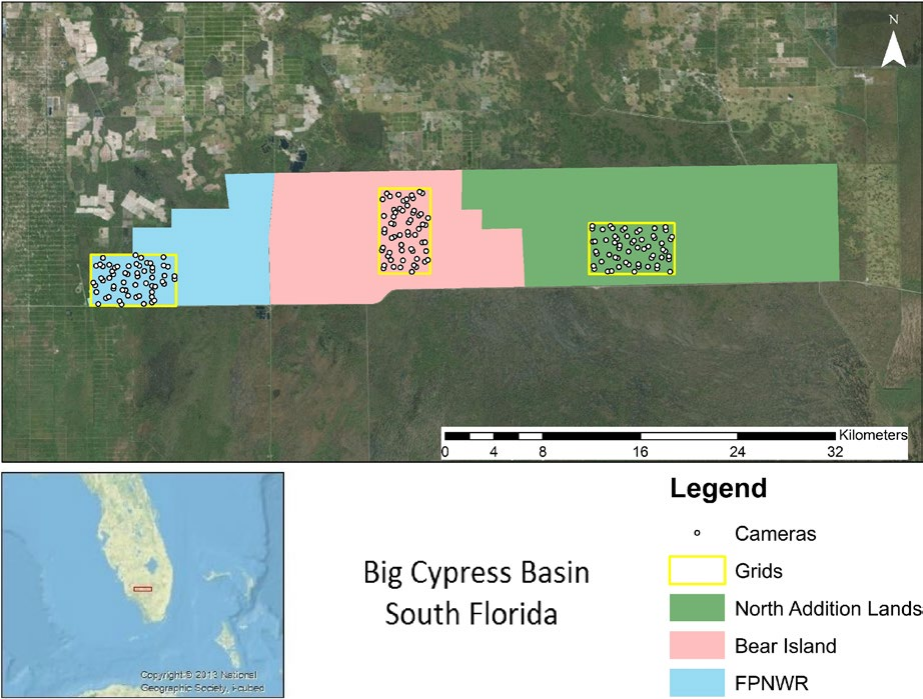
The research site was located in the Big Cypress Basin of southwestern Florida. To estimate the effects of risk of predation by Florida panthers on white‐tailed deer behavior, we deployed 60 infrared‐triggered cameras in each of three grids. Grids were separated by ≥13 km and were located in the Florida Panther National Wildlife Refuge and the Bear Island and North Addition Lands units of the Big Cypress National Preserve

The study occurred on the adjacent Florida Panther National Wildlife Refuge (FPNWR) and the Bear Island (BI) and Northeast Addition Lands (AL) units of Big Cypress National Preserve (BCNP) (Figure 1). The areas of FPNWR, BI, and AL encompassed approximately 100, 190, and 271 km^2^, respectively. Public accessibility differed among sites. Bear Island contained a network of off‐road vehicle (ORV) trails for public use by permit, and licensed hunting was permitted. Similarly, AL allowed public recreational access, but prohibited ORV access and limited issuance of hunting permits. The FPNWR prohibited public access, although it contained an extensive network of ORV trails to facilitate management activities. All sites contained ORV trail networks; however, the intensity of vehicular traffic on and maintenance of trails was variable. For example, regulation restricted vehicular access in AL to authorized administrative personnel who utilized the trail network infrequently for maintenance and wildfire containment purposes.

### 2.2 Study species

Because deer activity is closely linked to reproductive stage, we organized our study in the context of biological seasons of deer in the BCB (Richter & Labisky, 1985). Camera trap data indicated a broad window of fawning across most of February and March (Chandler et al., 2018); thus, we designated these months as the fawning season. This timescale (February–March) was chosen to appropriately characterize behaviors leading up to fawning, such as fawning site selection, while including the time period over which the majority of fawns were born. Because most fawns on our study site were born by the end of March and nearly all bucks had initiated antler growth by this point, we designated April–June 2015 as the fawn‐rearing and antler growth season (hereafter rearing). Rearing is an energetically expensive time for reproductive females as fawns grow and lactation peaks (Clutton‐Brock, 1982; Oftedal, 1985; Pekins, Smith, & Mautz, 1998). Similarly, males invest in antler development and body growth during this period because antler size and body mass are positively correlated with dominance and reproductive opportunity (Townsend & Bailey, 1981). We designated July as the prerut when males exhibit hyperphagy, increased activity, and increased antler sparring in preparation for conspecific competition. Given the relatively broad fawning window, some breeding occurred through August and September; however, peak breeding, or rut, behavior occurred in mid‐August to late August. Thus, we designated August as the rut. This is a stressful time for males as they forage minimally and maximize mate searching behaviors. Following the rut, males enter a recuperation phase known as the postrut (September–October). During this biological season, bred females are in the earliest stages of gestation. While the third trimester witnesses a peak in energetic demand for females, this period is partially included in the early fawning season. The majority of the period between the postrut and fawning seasons, or gestation, requires relatively low reproductive energetic investment. Thus, we did not consider gestation in analyses.

### 2.3 Camera trap array

In January 2015, we deployed 180 remote‐sensing infrared‐triggered cameras (HCO Outdoor Products, Norcross, GA, USA) without bait or any attractant across the study area in three grids containing 60 cameras each (Figure 1). The grids were placed at the same latitude and spaced by ≥13 km longitudinally. We placed 40 cameras in each grid on ORV trails and the remaining 20 approximately 250 m from the trail. We determined on‐trail camera trap (henceforth, trap) locations by overlaying aerial photography with 700 m^2^ grid cells using ArcGIS 10.2 (Environmental Systems Research Institute, Redlands, CA, USA) and placing traps near the center of cells to maintain an approximate distance of 700 m between on‐trail traps. We positioned on‐trail traps on the closest suitable tree and oriented each perpendicular to the trail. To maximize probability of detection of animals at off‐trail traps, we deployed traps on the most well‐defined wildlife trail or habitat edge within 50 m of the selected point. We positioned cameras approximately 0.30 m above ground, oriented either north or south, and adjusted height according to surface water levels to avoid inundation. Because camera traps were not baited, cameras were programmed to record images with no delay between sequential triggering events. We visited traps at approximately 30‐day intervals for data retrieval and camera maintenance, and vegetation was cleared as needed to minimize false‐triggering of cameras.

### 2.4 Data preparation and analysis

To maximize independence of detections, we sorted records chronologically by camera and omitted records with the same sex, age, and species class as the previous record from analysis if the time from the previous record was <6 min. We determined this threshold by filtering the data at 1‐min intervals and visually inspecting the mean difference in time between images at each thinning interval. The resulting curve indicated a rapid decrease in rate of change in the mean interval when images separated by 5 min or less were omitted. This procedure improved independence of detections by removing sequential images of lingering individuals. We then classified detections based on biological seasons and characterized each as either diurnal (between sunrise and sunset) or nocturnal (between sunset and sunrise). Package maptools (Bivand & Lewin‐Koh, 2015) in Program R (R Development Core Team, 2014) were used to determine daily sunrise and sunset times associated with the coordinates of the centroid of our study area.

We evaluated the effects of panther predation risk on adult deer activity patterns using the camera trap data. Neonate detections were omitted as they were considered naïve to risk and their activity dependent on maternal activity. We estimated predation risk by modeling panther activity patterns to predict when and where adult deer were likely to encounter a panther. We analyzed count data of male deer, female deer, and panthers at each camera using Poisson generalized linear mixed models (GLMM) with a log link. The response variable (*y_ijk_*) was the number of detections at each camera (*i* = 1, … 180)*,* during each time period (*j* = 1,2; for diurnal and nocturnal) and biological season (*k* = 1, … ,5; for fawning, rearing, prerut, rut, and postrut). Explanatory variables included trail (i.e., on‐ and off‐trail), time, and biological season. We fit GLMMs for each sex of deer and a single model for both sexes of panther. We constructed four candidate models representative of specific hypotheses, and we used AIC for model selection. Candidate models included various combinations of the main effects of trail, time, and biological season as well as 2‐way interactions of each. We hypothesized that time and biological season would interact such that deer detection rates would be greater at high‐risk times during biological seasons of reproductive importance. Similarly, we hypothesized that trail and season would interact such that deer detection rates would be greater in high‐risk places during biological seasons of reproductive importance. The number of camera hours varied among scenarios due to variable season and day length (e.g., nocturnal on‐trail during the fawning season) and among cameras due to camera failure, which we accounted for by using log(camera hours) as an offset in the GLMMs. As a result, the estimates can be interpreted as the number of detections per hour. We modeled variation among cameras using camera‐specific random effects. Due to difficulty of deriving asymptotic standard errors from linear models including random effects, we calculated 95% confidence intervals (CI) for detection rates via parametric bootstrapping, and deemed detection rates of bucks, does, and panthers significantly different when CIs for differences in means did not include zero. We conducted detection rate analyses in program R using package lme4 (Bates, Mächler, Bolker, & Walker, 2015).

To test for differences in activity overlap of deer with panthers, we calculated the coefficient of overlap in activity patterns of male and female deer with panthers using nonparametric kernel density estimation of detection times (Ridout & Linkie, 2009). We employed nonparametric bootstrapping to calculate confidence intervals for estimates of activity overlap. We estimated sex‐specific deer–panther activity overlap for every combination of trail (i.e., on, off) and biological season (i.e., fawning, rearing, prerut, rut, and postrut). We identified significant differences in activity overlap using CIs in the same manner as described for detection rates. We conducted activity pattern overlap analyses in program R using package overlap (Ridout & Linkie, 2009).

## 3 RESULTS

We recorded 1,058 independent detections of panthers, 1,799 independent detections of adult (i.e., ≥1 year of age) male deer, and 2,624 detections of adult female (i.e., ≥1 year of age) deer from February to October 2015. At the diel timescale, only 28% (*n* = 296) of panther detections occurred during diurnal periods. Spatially, 91% (*n* = 966) of panther detections occurred at on‐trail traps. Sixty‐five percent (*n = *1,177) of male deer detections were diurnal and 65% (*n = *1,175) occurred at on‐trail traps. Seventy‐one percent (*n = *1,862) of female deer detections occurred during diurnal hours, while 60% (*n = *1,565) of adult female deer detections occurred at on‐trail traps. However, only 11% (*n = *279) of female deer detections occurred on‐trail during nocturnal hours.

### 3.1 Detection rates

The most supported model for panthers and both sexes of deer included trail × time, trail × season, and season × time interactions (Table 1). We observed an interactive effect of trail and time on the rate of detection of panthers. This interaction is evident in an 875% increase in detection rates from diurnal off‐trail traps during the rut (0.24, 95% CI: 0.15–0.36; detections/1,000 hr) to nocturnal on‐trail traps in the fawning season (1.02, 95% CI: 0.80–1.24). We also observed a season *x* time interactive effect on detection rates of panthers with diurnal and nocturnal detection rates being highest during the fawning season at both on‐trail and off‐trail traps. The detection rate of panthers was greater on‐trail than off‐trail during both day and night across all seasons with the highest detection rates observed on‐trail at night during the fawning season (1.03, 95% CI: 0.82–1.28) and on‐trail at night during the rut (1.02, 95% CI: 0.80–1.24). The lowest panther detection rates occurred off‐trail during diurnal hours of the prerut (0.23, 95% CI: 0.13–0.34) and rut seasons (0.24, 95% CI: 0.15–0.36).

**Table 1:** Model selection results for models used to predict male and female (≥1 year old) white‐tailed deer and Florida panther detection rates at camera traps on the Big Cypress National Preserve and Florida Panther National Wildlife Refuge in Collier County, FL, USA (February–October 2015)

Model	Parameters	AICc	ΔAICc	AICc weight
*Male Deer*				
Trail:Season + Trail:Time + Season:Time	17	12,601	0	1
Season:Time	11	12,803	202	0
Trail:Time + Season	9	12,926	325	0
Trail + Season + Time	8	12,935	335	0
*Female Deer*				
Trail:Season + Trail:Time + Season:Time	17	16,116	0	1
Trail:Time + Season	9	16,512	396	0
Season:Time	11	16,943	826	0
Trail + Season + Time	8	17,004	888	0
*Panther*				
Trail:Season + Trail:Time + Season:Time	17	5,307	0	1
Trail:Time + Season	9	5,354	47	0
Trail + Season + Time	8	5,384	77	0
Season:Time	11	5,410	103	0

**Figure 2:**
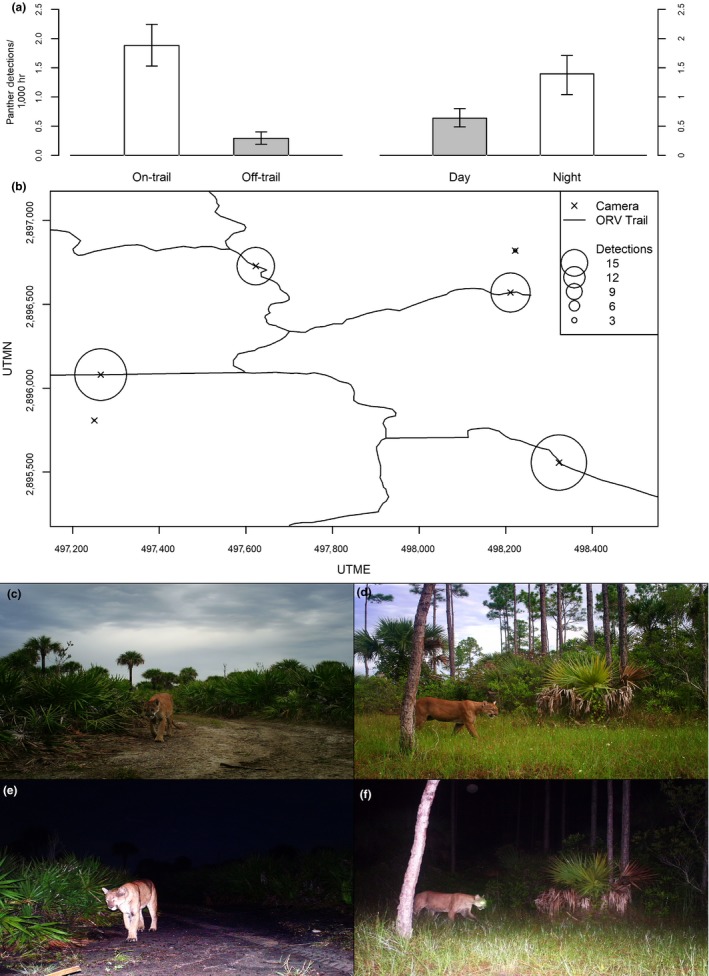
(a) Diurnal and nocturnal panther detections per 1,000 hours at on‐ and off‐trail camera traps February–October 2015, Big Cypress Basin, Florida. Error bars indicate bootstrapped 95% confidence intervals. (b) Detections of panthers at a subset of camera traps on the study site. Images depict examples of (c) diurnal on‐trail, (d) diurnal off‐trail, (e) nocturnal on‐trail, and, (f) nocturnal off‐trail panther detections

The difference between diurnal and nocturnal detection rates clearly identified nocturnal hours as periods of higher predation risk to deer (Figure 2). At the seasonal scale, panther activity varied little with the exception of increased diurnal activity during the fawning season. Spatially, panther detection rates were much higher at on‐trail traps than off‐trail, suggesting high risk of predation in the vicinity of trails. Thus, we considered deer activity in the context of spatially and temporally variable risk of predation. We classified diurnal and nocturnal periods as low‐ and high‐risk times, respectively, and we considered on‐trail and off‐trail locations as areas presenting respective high and low risk. Therefore, diurnal, off‐trail activity imposed the least risk and nocturnal, on‐trail activity imposed the greatest risk.

**Figure 3:**
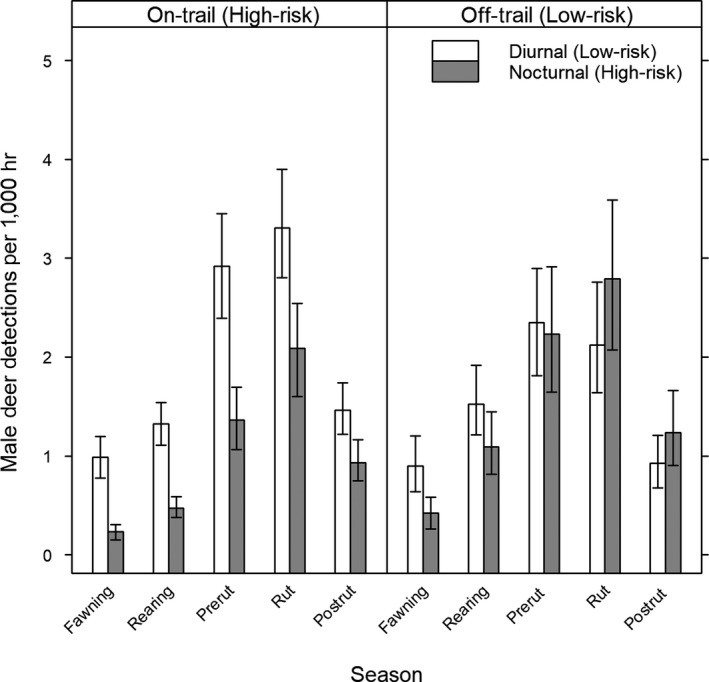
Diurnal and nocturnal male deer detections per 1,000 hours at on‐ and off‐trail camera traps by biological season, Big Cypress Basin, Florida. Fawning includes February–March 2015, rearing includes April–June 2015, prerut includes July 2015, rut includes August 2015, and post‐rut includes September–October 2015. Error bars indicate bootstrapped 95% confidence intervals

For male deer, we observed significant interactive effects of trail and time as well as time and season on detection rates (Figure 3). In high‐risk areas at low‐risk times, detection rates (detections/1,000 hr) of males were lowest during the fawning season (0.98, 95% CI: 0.77–1.20) and peaked during the rut (3.31, 95% CI: 2.80–3.90). Detection rates were greater at low‐risk times than high‐risk times across all seasons. In high‐risk areas at high‐risk times, male activity was lowest during the fawning season (0.23, 95% CI: 0.15–0.31) and increased each season through the rut (2.09, 95% CI: 1.60–2.54) then decreased during the postrut (0.93, 95% CI: 0.75–1.16). In low‐risk areas at low‐risk times, activity of males was lowest during fawning (0.90, 95% CI: 0.64–1.20) and peaked during prerut (2.35, 95% CI: 1.81–2.90) and rut (2.12, 95% CI: 1.64–2.76). In low‐risk areas, male activity during low‐risk times was greater than during high‐risk times during fawning and rearing, but there was no difference during any other season.

**Figure 4:**
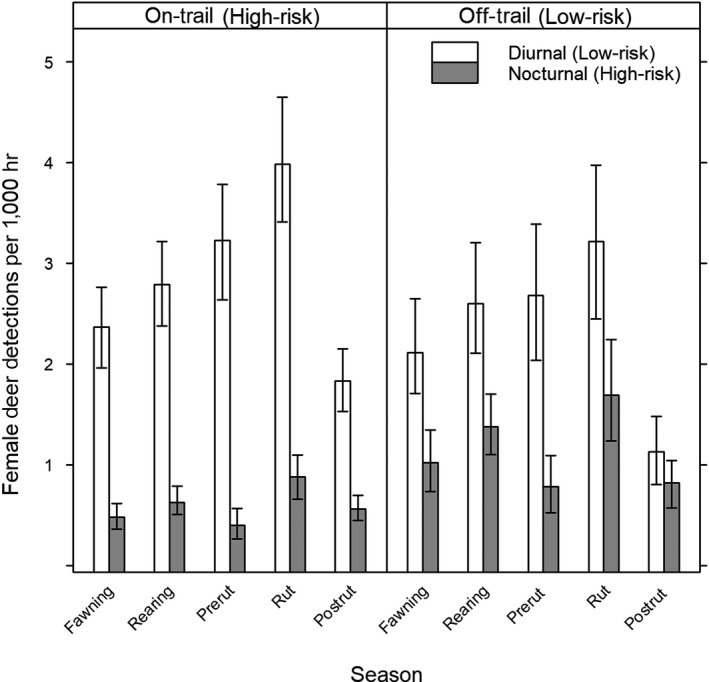
Diurnal and nocturnal female deer detections per 1,000 hours at on‐ and off‐trail camera traps by biological season, Big Cypress Basin, Florida. Fawning includes February–March 2015, rearing includes April–June 2015, prerut includes July 2015, rut includes August 2015, and post‐rut includes September–October 2015. Error bars indicate bootstrapped 95% confidence intervals

We observed interactive effects of trail and time as well as season and time on detection rates of female deer (Figure 4). Detection rates of females were greater at low‐risk times across all seasons regardless of location. The greatest female detections rates occurred in high‐risk areas at low‐risk times during the rearing (2.79, 95% CI: 2.38–3.22), prerut (3.23, 95% CI: 2.64–3.79), and rut (3.98, 95% CI: 3.41–4.65) seasons. However, detection rates of females at high‐risk times were greater in low‐risk areas through all seasons.

### 3.2 Activity overlap

**Figure 5:**
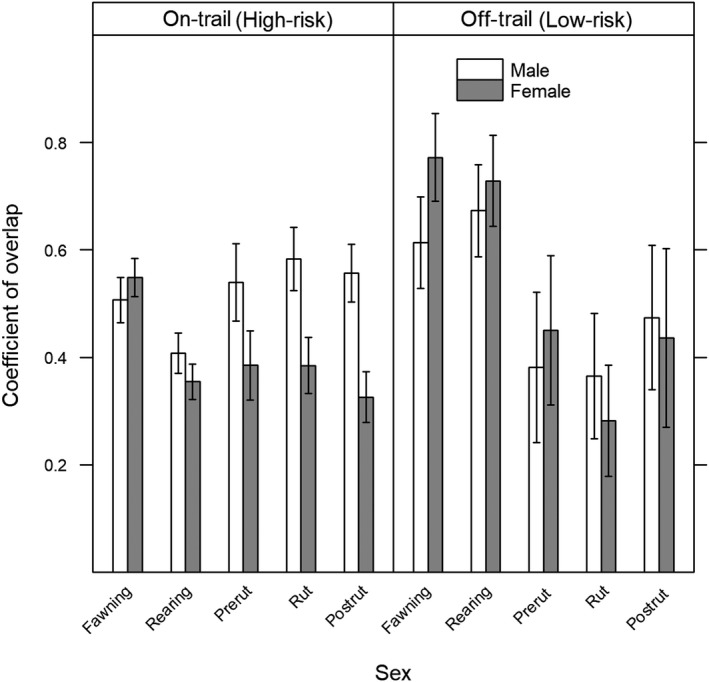
Overlap of male and female deer activity patterns with panther activity at on‐ and off‐trail camera traps by biological season, Big Cypress Basin, Florida. Fawning includes February–March 2015, rearing includes April–June 2015, prerut includes July 2015, rut includes August 2015, and postrut includes September–October 2015. Error bars indicate bootstrapped 95% confidence intervals

We observed significant effects of trail and season on the coefficient of overlap of males and females with panthers (Figure 5). In low‐risk, off‐trail areas, the sexes only differed in overlap with panthers during the fawning season when female‐panther overlap was greater. However, the sexes differed in overlap with panthers during all seasons in high‐risk, on‐trail areas where females overlapped with panthers more during the fawning season, and male‐panther overlap was greater during the rearing, prerut, rut, and postrut seasons. We also observed seasonal differences in overlap within the sexes. In low‐risk areas, activity overlap was greater during fawning and rearing than prerut, rut, and postrut for both sexes, and female‐panther overlap was lower during the rut than any other season. In high‐risk, on‐trail areas, female‐panther overlap was greatest during the fawning season while male‐panther overlap was greatest during the rut. Within the sexes, we also observed effects of spatial variation in risk of predation on deer–panther overlap; female‐panther overlap was lower in high‐risk areas than low‐risk areas during fawning and rearing, while male‐panther overlap in high‐risk areas was lowest during rearing and greatest during rut.

## 4 DISCUSSION

Our results provide strong correlative evidence that risk of predation by panthers induces white‐tailed deer activity patterns that are substantially different from activity patterns in other parts of their range where panthers do not occur. Activity patterns of deer vary based on geographical, physiological, and environmental factors; however, peaks in activity during crepuscular hours are ubiquitous across the species’ range (Kammermeyer & Marchinton, 1977; Beier & McCullough, 1990). Increases in nocturnal activity of deer exposed to human hunting pressure are also well documented (Kilgo, Labisky, & Fritzen, 1998; Kilpatrick & Lima, 1999; Little et al., 2015; Webb, Gee, Strickland, Demarais, & DeYoung, 2010). In our system, our results suggest that both sexes of deer displayed preference for diurnal activity and support the “risky times hypothesis,” which predicts that prey respond to temporal variation in risk. Our results suggest that males engaged in riskier, nocturnal activity more than females, which may be attributed to their inability to forgo activity during periods of high risk while meeting energetic requirements for maintaining reproductively competitive body mass. Conversely, female detection rates suggest a strong aversion to nocturnal activity.

In addition to sex‐specific responses of deer to temporal variation in risk of predation, we also found sex‐specific responses to spatial variation in risk, which support the “risky places hypothesis.” In our study, on‐trail detection rates of panthers were up to 875% greater than off‐trail rates. This difference in space use by panthers allowed us to test for the effects of spatial variation in risk of predation, which revealed apparent avoidance of high‐risk areas by deer, particularly at high‐risk times. The ability of prey to perceive spatial variation in risk and alter their behavior accordingly has been demonstrated across taxa (Sih, 1980; Brown, 1999). Such behavioral decisions made under the risk of predation are the process by which the “landscape of fear” is shaped (Laundré et al., 2001). For example, Altendorf, Laundré. Gonzalez, and Brown (2001) demonstrated that mule deer (*O. hemionus*) sympatric with cougars (*P. c. cougar) *of western North America perceive forest edges as high‐risk areas using giving‐up densities. Conversely, reintroduction of wolves shifted habitat selection by elk from open habitat types to closed‐canopy habitats (Creel, Winnie, Maxwell, Hamlin, & Creel, 2005).

We hypothesized that males would be more active in high‐risk scenarios than females and that the sexes would be most risk prone during times of relative reproductive importance (i.e., rut and rearing for male and female deer, respectively). Predation risk has been suggested as a driver of behavioral variation among male and female deer (Ruckstuhl & Neuhaus, 2000) as sex‐specific energetic demands associated with reproductive success require trade‐offs between safety and energetic intake (Main, Weckerly, & Bleich, 1996; Ruckstuhl & Neuhaus, 2000). We observed increased exposure to high‐risk scenarios for male deer during seasons of high reproductive importance. Our results indicate an increase in high‐risk male activity leading up to and during the breeding season when males seek out and compete for mates. However, females did not appear to increase risk exposure as predicted during the fawning and rearing seasons relative to other seasons.

While detection rates indicate no increased female risk exposure during fawning and rearing, our activity overlap results supported the hypothesis that differing requirements for reproductive success explain the behavioral differences between the sexes. Female deer experience the greatest temporal overlap in activity with panthers during fawning at both on‐ and off‐trail traps, but relatively high diurnal activity of panthers during fawning may have contributed to increased overlap. Relatively high female overlap with panthers during the rearing season, particularly off‐trail, may be explained by increased female nocturnal activity. Females experience a relatively short but intense increase in energetic demand associated with lactation, but can otherwise energetically afford the relative safety of decreased activity. Conversely, male fitness is positively correlated with body mass, which requires a greater frequency of high‐risk foraging bouts.

A growing body of evidence suggests that anthropogenic disturbance may affect predator–prey systems with adverse consequences for prey populations (DeGregorio, Weatherhead, & Sperry, 2014; Stuart‐Smith, Bradshaw, Boutin, Hebert, & Rippin, 1997). Caribou (*Rangifer tarandus*) mortality sites associated with wolves and human hunting were closer to roads than random caribou telemetry locations (James & Stuart‐Smith, 2000). Our results provide strong evidence that deer on our study site perceive ORV trails as high‐risk areas and reserve activity in those areas for low‐risk times to minimize probability of encounters with panthers, which we detected disproportionately on‐trail. Relatively high detection rates of panthers at on‐trail traps suggest that ORV trails may facilitate efficient movement of panthers across the southwestern Florida landscape.

Following reintroduction of wolves to the Greater Yellowstone Ecosystem (Wyoming, USA) in the mid‐1990s (Laundré et al., 2001), shifts in elk behavior, such as alterations in vigilance rates and space use, demonstrated the profound behavioral impacts predators can have on prey (Creel et al., 2005; Halofsky & Ripple, 2008; Kauffman et al., 2007; Winnie, 2012). Our results afford the unique opportunity for comparison of postrestoration behavioral effects of canid versus feline predators on North American cervid species. Unlike (Creel et al., 2005) who provided support for the “risky places hypothesis,” but found none for the “risky times hypothesis,” our results support both. These differences in findings may be a function of predator hunting mode. Ambush predators, such as panthers, should exact a greater magnitude of antipredator responses than cursorial predators, as there likely are habitat cues associated with ambush predators while encounters with cursorial predators are less predictable (Kauffman et al., 2007). Similarly, our results suggest highly predictable temporal patterns of activity for panthers, which suggests that darkness may serve as a temporal cue of risk. Although our study lacks the design to causally link panthers to spatiotemporal shifts in deer activity, we suggest future research focus on comparing deer activity in the presence and absence of predators to further develop our understanding of the impacts of predator hunting mode on prevalence and relative magnitude of behavioral risk effects. Our results provide support for the hypothesis that predation risk shapes the spatial distribution and temporal activity patterns of prey populations (Brown, Laundré, & Gurung, 1999; Laundré, 2010) as well as evidence that white‐tailed deer perceive spatial and temporal variability in risk and alter their behavior to mitigate exposure to that risk.

## CONFLICT OF INTEREST

None declared.

## AUTHOR CONTRIBUTIONS

Daniel A. Crawford collected field data, participated in data analysis, and drafted the manuscript; Michael J. Cherry was a co‐principle investigator on the study, participated in the design of the study, participated in the statistical analyses, coordinated the study, and helped draft the manuscript; Brian D. Kelly collected field data; Elina P. Garrison was a co‐principle investigator on the study, coordinated the study, and participated in the design of the study; David Shindle was a co‐principle investigator on the study and participated in the design of the study; L. Mike Conner was a co‐principle investigator on the study, participated in the design of the study, and helped draft the manuscript; Richard B. Chandler was a co‐principle investigator on the study, participated in design of the study, participated in statistical analysis, and helped draft the manuscript; Karl V. Miller was a co‐principle investigator on the study, participated in design of the study, and helped draft the manuscript. All authors gave final approval for publication.

## Data Availability

Supporting data for analyses may be found in the Dryad Data Repository (https://doi.org/10.5061/dryad.qn55s).
